# Community Treatment Orders in Australia in the Context of International Literature: A Narrative Review of Consumers, Families and Health Professionals' Perspectives

**DOI:** 10.1111/inm.70061

**Published:** 2025-05-09

**Authors:** Madeleine Harvery, Rosamund Harrington, Matthew Morgan, Elisa Yule, Shelley Wright

**Affiliations:** ^1^ School of Allied Health, Faculty of Health Sciences Australian Catholic University Sydney New South Wales Australia; ^2^ Thomas More Law School Australian Catholic University Brisbane Queensland Australia

**Keywords:** coercion, community mental health services, involuntary commitment, mental health recovery, principle‐based ethics

## Abstract

A community treatment order (CTO) sets out legal conditions requiring a person experiencing mental illness to receive treatment and medication while living in the community. Although CTOs are used internationally in most high‐income nations, CTO use in Australia is very high compared with world standards. CTOs are a contentious issue in mental health practice. While they aim to protect both consumers and society, they often interfere with personal freedom and trust between consumers and mental health professionals. International and Australian research is inconclusive about the effectiveness of CTOs in achieving their clinical aims. Australian research overwhelmingly reports consumers' experience with CTOs as coercive and distressing. Families generally support CTOs but are unhappy with certain aspects of the process. Consumers and families' feelings about CTOs often depend on how much they trust the mental health team, the support they receive, and the relationship between the consumer and mental health professional. Health professionals experience significant challenges balancing therapeutic care with their role carrying out CTOs. Local treatment culture, problems in the mental health system and staff attitudes also influence how CTOs are used. There is a lack of recent and high‐quality guidance to inform CTO decision‐making for mental health professionals. This perspective paper is presented as a narrative literature review, which proposes that if CTOs are used in clinical practice, close consideration towards how and why they are implemented is warranted.

## 1 Background

A community treatment order (CTO)—referred to internationally as compulsory outpatient treatment or mandatory outpatient commitment—specifies conditions under which a person experiencing mental illness must accept mental health treatment and medication in the community (Light 2019). Internationally, their use generally requires a person to: (1) have a mental health condition, (2) be at risk of harm to themself or others, and (3) refuse to adhere to treatment (Churchill et al. 2007). In Australia, CTOs are granted by mental health tribunals and implemented by public mental health services. Nonadherence to the terms of a CTO can activate involuntary return to hospital for assessment, medication and potential readmission (Barnett et al. 2018).

Although CTOs are used internationally in most high‐income nations, Australia has very high rates of CTO use by world standards (Light 2019). Comparing the use of CTOs between jurisdictions both within Australia and internationally is challenging given disparities in data collection, reporting systems, data availability and comparability (Light 2019). However, in a review of the data on international CTO use, Lawton‐Smith (2005) reported CTOs appear to be rising internationally and that there is limited published international data on the number of people on CTOs.

Since the deinstitutionalisation of mental health services and the introduction of CTOs, their use has been contentious in international mental health practice. Tensions exist between the duty of care by mental health services, managing perceived risks to society, impact on the therapeutic relationship and intrusion into the liberty of a person with decision‐making capacity who has not committed a crime (Rugkåsa and Burns 2017). Additional concerns emerge with evidence suggesting CTOs are disproportionately applied to minority populations, with First Nations Australians and people from culturally and linguistically diverse [CALD] backgrounds being at an increased likelihood of involuntary community treatment (Kisely and Xiao 2018; Kisely et al. 2020). For example, Moss et al. (2019) compared CTOs in a CALD population with a non‐CALD population in Queensland, Australia, finding involuntary treatment almost tripled when an interpreter was used (Moss et al. 2019). These findings may reflect the intersection of mental health stigma and race‐based discrimination, reluctance by people from CALD backgrounds to seek help until symptoms are severe or issues relating to the accurate diagnosis of mental illness cross‐culturally (Turan et al. 2019).

Concerns about the particularly high rate of CTOs in Australia have become prominent in recent years, in part because CTOs arguably do not adhere to the United Nations *Convention on the Rights of Persons with Disabilities* (UNCRPD) (2006), which Australia ratified in 2008. The UNCRPD marked a revolution in international human rights, providing the first international framework to set out the rights of people with disabilities (Maylea and Hirsch 2017). By ratifying the UNCRPD, states have committed to reforming their domestic mental health law to not discriminate based on disability and chronic mental illness. Under the UNCRPD, involuntary treatment of mental illness threatens several human rights, such as the right to ‘equal recognition before the law’ (Article 12) and the right to ‘freedom from torture or cruel, inhuman or degrading treatment or punishment’ (Article 15). Given individuals with capacity have the right to refuse treatment in most circumstances, and CTOs permit treatment against a person's will solely due to disability, CTOs violate the right to refuse treatment (Newton‐Howes and Ryan 2017).

CTOs emerged in Australia in the state of Victoria in 1986 as a less restrictive alternative to institutionalised involuntary care, aligning with shifts in mental health policy for treating people in the least restrictive environment (Rugkåsa and Burns 2017). The introduction of CTOs was also a response to concerns from mental health professionals regarding the impacts of deinstitutionalisation, specifically the emergence of ‘revolving door patients’; a population viewed as being at high risk of ceasing medications, failing to keep outpatient appointments, and subsequently being at high risk of relapse and rehospitalisation (Rugkåsa and Burns 2017). In addition, public fear regarding rare high‐profile international acts of violence committed by people with mental illness now living in the community, combined with system pressures on acute psychiatric hospital beds, resulted in development of legislation to allow involuntary community treatment (Hsieh et al. 2017). Thus, the use of CTOs is characterised by a conflict of opposing ethical interests which continues to be a contentious issue in mental health practice internationally and in Australia. The aim of this perspective paper was to therefore examine the experiences of consumers, families and mental health professionals towards CTOs in Australian mental health practice settings.

## 2 Design

To contextualise these experiences, the authors initially present a discussion around the efficacy of CTOs internationally and nationally and argue that the ongoing and increasing use of CTOs in Australia is concerning given the unclear and insufficient evidence base. This perspective paper is presented as a narrative literature review from three different stakeholder perspectives and aims to critically examine the factors influencing stakeholder experiences and patterns of CTO use.

## 3 Methods

The search strategy was conducted on 27th January 2023 and rerun on 8th April 2025 in APA PsychINFO, CINAHL Complete, Embase and MEDLINE Complete. The key search terms used were (1) compulsory or involuntary or mandat* or coerciv*, (2) treatment or commitment or order*, (3) community or civil or outpatient* or out‐patient*, and (4) “outpatient civil commitment” or “out‐patient civil commitment.” Search terms 1, 2 and 3 were searched together using proximity Boolean operators to locate articles using the various terminologies adopted internationally to refer to CTOs. Articles were selected for relevance by reviewing the title and abstract, and the reference lists of relevant selected articles were also searched and reviewed. Included articles were published in English and no date limits were applied. Initial searches were made purposely broad to capture a wide range of issues relating to CTOs. As the narrative review progressed, the scope was narrowed to focus more specifically on consumer, family and health professional experiences of CTOs in Australia. Articles with a focus on persons under the age of 18 or over the age of 65 were excluded, as in NSW these groups are managed by specialised mental health services, such as an Early Psychosis service for young people, and have additional health needs affecting capacity such as dementia. Articles with a focus on forensic CTOs were also excluded, as forensic CTOs are dealt with under different legislation, and a person on a forensic CTO is living in a correctional facility rather than in the community. The grey literature was also searched, which involved hand searching the reference lists of relevant articles and searching Google Scholar to locate information pertaining to UN Conventions and Australian state and territory legislation. A quality appraisal of this narrative review was guided by the SANRA, the Scale for the Assessment of Narrative Review Articles (Baethge et al. 2019).

## 4 The Context of CTOs: Use and Efficacy

### 4.1 Use of CTOs Internationally

Legal regimes for involuntary community mental health care currently exist in approximately 75 jurisdictions internationally (Rugkåsa and Burns 2017). Beginning in the 1980s, CTOs were introduced in the United States and Canada (Churchill et al. 2007), and then in the late 1980s and early 1990s in Australia and New Zealand (J. Dawson 2005). CTOs have also been introduced in many European countries and some Asian countries (Rugkåsa and Burns 2017). Globally, CTOs differ in relation to duration, the requirement of nonadherence to treatment or hospital readmissions, and whether they can be commenced in an inpatient or community‐based setting (Dawson 2005).

Due to jurisdictional disparities in CTO use, the international evidence of the efficacy of CTOs is largely unclear. A small body of literature exists using randomised control trials (RCTs) to examine the effectiveness of CTOs on a variety of outcomes, including treatment and medication adherence, quality of life, rehospitalisation and criminality (Burns et al. 2013; Steadman et al. 2001; Swartz et al. 1999). Swartz et al. (1999) evaluated the effectiveness of CTOs compared to voluntary treatment in reducing rehospitalisation in North Carolina, USA. For CTOs longer than 180 days, a significant effect was observed with a reduced number of hospital admissions; however, only when combined with greater intensity of community treatment (OR = 0.64, 95% CI = 0.46–0.88, *p* ≤ 0.01). Steadman et al. (2001) examined the effect of a three‐year CTO pilot programme in New York, USA, on rehospitalisation, quality of life, symptomatology, arrest, treatment adherence and perceived coercion. Participants were randomised to either CTO treatment or voluntary community treatment. There were no between‐group differences on any clinical outcomes, and participants rated perceptions of their quality of life and level of coercion as similar.

The Oxford Community Treatment Order Evaluation Trial (OCTET) study in the United Kingdom followed participants randomised to either a CTO upon hospital discharge or a less intensive supervised discharge (Burns et al. 2013). Participants were followed over 12 months and were primarily measured on whether they were admitted to hospital during the follow‐up period. No between‐group differences were observed on clinical outcomes or level of community contact. In the 3‐year follow‐up study, no between‐group differences were observed in numbers of consumers readmitted, number or duration of readmissions or time to first readmission (Burns et al. 2015). One significant finding was that longer CTOs were associated with subsequent disengagement, bringing into question whether a less restrictive approach may be more beneficial long‐term. The findings of these RCTs suggest there is limited high‐quality evidence to demonstrate the effectiveness of CTOs, with the authors concluding that the restrictive nature of CTOs is not outweighed by evidence of favourable outcomes.

Several systematic reviews and meta‐analyses in Australia, the United Kingdom and Norway support the conclusions of RCTs, determining that compared with standard voluntary treatment, CTOs have no significant effect on hospital readmissions or bed days (Barnett et al. 2018; Kisely et al. 2007, 2021; Kisely and Hall 2014; Maughan et al. 2014), treatment adherence (Barnett et al. 2018), psychiatric symptoms or social functioning (Kisely and Hall 2014; Kisely, Campbell, et al. 2017; Kisely, Siskind, et al. 2017), quality of life or satisfaction with care (Kisely, Campbell, et al. 2017; Kisely, Siskind, et al. 2017), health service use (Kisely, Campbell, et al. 2017; Kisely, Siskind, et al. 2017; Maughan et al. 2014), aggression or criminal behaviour (Kisely et al. 2025) or long‐term treatment adherence post‐CTO (Cossu et al. 2024). A systematic review conducted in Australia found some benefit where people on CTOs were less likely than controls to be victims of crime (RR = 0.50, CI = 0.31–0.80), however, the authors note ‘it is unclear whether this benefit is due to the intensity of treatment or its compulsory nature’ (Kisely, Campbell, et al. 2017; Kisely, Siskind, et al. 2017, 2). Kisely et al.'s (2021) study, a systematic review only including studies in Australia and New Zealand, also found evidence of lowered mortality for people on CTOs (log‐adjusted RR = −0.28, 95% CI = ‒0.41, −0.15) and an increased benefit longer‐term of reducing readmissions, but only after at least 2 years of use (IRR = 0.72, 95% CI = 0.60–0.85, *p* < 0.0005). Barnett et al. (2018) commented on the significant variability in study quality, stating that bias frequently related to poor comparability between CTO and control participants.

Notably, the use of RCTs to examine CTOs has been both criticised and defended (O'Reilly and Vingilis 2018), with some academics having called for ‘expansion of the CTO evidence base to include not only RCTs but also other study designs which may be better suited to capture their real‐world effectiveness’ (Swartz and Swanson 2015, 852). It is argued that RCTs analysing CTOs resulted in small‐scale studies of highly specific populations, which had difficulty fitting complex community interventions into the rigid fidelity protocol of RCTs (O'Reilly and Vingilis 2018). Others argued that RCTs have not included the correct group of consumers likely to benefit from a CTO or have not applied the CTO appropriately (Curtis 2014). Specific to the OCTET study, Curtis (2014) raised selection bias concerns, stating ‘the psychiatrists who volunteered to participate would be largely those who were doubtful of the value of CTOs and hence would be happy to submit patients for randomisation’ (p. 37). Thus, the study design may have recruited psychiatrists who were either ambivalent or unclear about the usefulness of CTOs. These concerns raise questions regarding whether RCTs measuring CTO efficacy can realistically lead to widely generalisable evidence that is relevant across diverse communities, treatment programmes and mental health systems (Swartz and Swanson 2015).

### 4.2 Use of CTOs in Australia

Australian mental health legislation operates on a state/territory‐based system, with each of Australia's eight states and territories (Australian Capital Territory [ACT], New South Wales, Northern Territory, Queensland, South Australia, Tasmania, Victoria, Western Australia) governing their own mental health acts. CTOs exist in all Australian jurisdictions, with some CTO programmes operating for decades. However, the processes regarding CTOs vary considerably between jurisdictions. For example, the *Mental Health Act 2016* (Qld) makes specific mention that ‘a person may have capacity to consent to be treated even though the person decides not to receive treatment’ (s. 14), whereas the *Mental Health Act 2007* (NSW) has no such mention. The proportion of CTOs used across jurisdictions also has significant variation. For example, 1.7% use in Western Australia compared with 37.6% in ACT, which most likely reflects differences in legislation (Australian Institute of Health and Welfare 2018). Thus, an important factor in the discussion regarding CTOs is the complexities in comparing their use across different legal and mental health systems, both nationally and internationally (Churchill et al. 2007).

The ongoing and increasing use of CTOs in Australia is concerning given the insufficient evidence about their effectiveness, with case–control and cohort studies demonstrating inconsistent and conflicting findings. Recent observational studies in NSW and Victoria suggest positive effects of CTOs, including improved medication and service adherence (Segal 2020), lowered mortality via facilitated access to healthcare (Segal et al. 2017b), enhanced quality of life (Segal et al. 2017a), reduced likelihood of being a perpetrator or victim of crime (Segal et al. 2019) and delayed rehospitalisation (Harris et al. 2019). In a recent NSW‐based case–control study involving all persons receiving a CTO from 2004 to 2009 (*n* = 5548), the authors conclude that compared with pre‐CTO baseline and matched controls, individuals on CTOs had increased frequency of community‐based contacts (IRR = 1.35, 95% CI = 1.30–1.41, *p* < 0.0005), thereby reducing the likelihood of readmission (OR = 0.89, 95% CI = 0.81–0.99, *p* < 0.05) and delaying time to readmission (IRR = 1.35, 95% CI = 1.23–1.48, p < 0.0005) (Harris et al. 2019). These findings are supported by other case–control studies in Victoria (Segal and Burgess 2006b, 2006c) finding CTOs are effective in reducing hospital readmissions. However, despite the reduction in likelihood of readmission in the Harris et al. (2019) study, a CTO needed to be in place for more than 2 years to reduce bed days. Other observational research in Victoria (Segal and Burgess 2006a) and Western Australia (Kisely et al. 2004) suggests conflicting results, concluding that CTOs are not effective in reducing hospital readmissions, bed days or treatment costs. Therefore, there remain unclear and conflicting findings on CTO effectiveness from observational studies, resulting in difficulties in generalisability and applicability across diverse mental health settings.

Many observational studies seeking to establish CTO effectiveness are contested on methodological grounds due to short or absent follow‐up periods, poor comparability between CTO and control participants and exclusion of people subject to multiple CTOs (Barnett et al. 2018). For example, two research studies conducted in Victoria (Segal et al. 2017a, 2017b) have been critiqued based upon the unusual inclusion of patients with dementia, potentially significantly impacting mortality outcomes (Kisely, Campbell, et al. 2017; Kisely, Siskind, et al. 2017). In addition, for studies that include follow‐up periods, most benefits during the CTO period were not sustained after their conclusion. For example, a large register‐based study in Victoria found although extended CTOs were associated with reduced hospitalisation and increased use of community mental health services, these outcomes were not sustained after the CTO conclusion (Segal and Burgess 2006b, 2006c). The outcomes of the Harris et al. (2019) study have also been critiqued in their applicability to the current mental health service context. Ryan (2019) notes that the study included data from 2004 to 2009, which is before any Australian legislation incorporated decision‐making capacity into using a CTO. Since 2013, every Australian jurisdiction (except the Northern Territory) has reformed legislation to prohibit or strongly discourage CTOs in individuals who have capacity to refuse (Callaghan and Ryan 2016). Ryan (2019) suggests ‘it is possible that CTOs are far more potent when their use is restricted to those who lack decision‐making capacity’ (p. 12), and that the CTO's benefits were limited in the Harris et al. (2019) cohort due to the inclusion of participants who may have had decision‐making capacity regarding their mental health treatment.

When considered broadly, many benefits derived from CTOs seen in observational studies may be more reflective of the benefits of increased community mental health contact, rather than benefits deriving from a CTO's coercive effect. Increased community contact is associated with reduced hospital admission in most case–control studies (Kisely et al. 2013; Segal and Burgess 2006b). People on CTOs in the Harris et al. (2019) study averaged 3.9 community mental health contacts per month, significantly more than the control group who received 2.5 contacts per month. In a Victorian case–control study, CTOs were associated with a 20% reduction in risk of noninjury‐related deaths, however the authors note these benefits were seen due to the CTO facilitating access to medical care (Segal et al. 2017b). In a Western Australian study investigating the effect of CTOs on mortality rates, any reduction in mortality was lost after adjusting for post‐CTO community mental health service contacts (Kisely et al. 2013). The authors concluded ‘this finding suggests that increased contact (potentially achievable through other means such as assertive community treatment) rather than the CTO itself was the key factor’ (Kisely, Campbell, et al. 2017; Kisely, Siskind, et al. 2017, 1322). The outcomes of these studies highlight the role of community mental health services in improving the identification and treatment of physical health concerns in the mental health population.

## 5 The Experience of CTOs

### 5.1 Consumer Experiences

A systematic review of international research spanning Canada, New Zealand, Norway, the United States and the United Kingdom indicates consumers hold differing perspectives about CTOs (Corring et al. 2017). Consumers' experiences of CTOs are connected to the perceived trustworthiness of health professionals and the quality of supports, and the relationship between consumer and health professional shapes how consumers interpret health professionals' behaviour and the restrictive interventions (Haynes and Stroud 2019; Stuen et al. 2015). Although consumers report varied experiences of CTOs, negative experiences are more frequently reported in the literature (Maylea et al. 2025). Consumers often experience CTOs as coercive and dehumanising (Nyttingnes et al. 2016), with the feeling of being seen only as ‘patient’ perceived to inhibit their capacity to take responsibility for their own recovery and wellbeing (Stensrud, Høyer, et al. 2015). Conversely, some Canadian research indicates consumers are ambivalent towards CTOs, expressing ‘being on a CTO was better than being in the hospital’ (Nakhost et al. 2019, 726). Other Norwegian research indicates consumers consider CTOs as beneficial to their recovery by providing a safety net and supporting their access to care (Stuen et al. 2015). Some consumers value the easier access to healthcare services (Stensrud, Høyer, et al. 2015), and others accept CTOs provided they receive other beneficial services, such as housing and financial assistance (Stuen et al. 2015). Corring et al. (2017) commented on the methodological limitations of the studies included in their systematic review, acknowledging that qualitative studies may recruit participants with more positive views about CTOs, due to the possibility that individuals who are the most dissatisfied with CTOs may view research as associated with the orders and thus be less likely to enter a study.

In Australia, recovery principles underpin the national framework for mental health services and workforce capability (Commonwealth of Australia 2013). Personal recovery is viewed as a journey towards gaining hope, increasing personal autonomy, finding purpose in life and growing positive self‐esteem (Damsgaard and Angel 2021). However, Australian research overwhelmingly reports consumers experience CTOs as coercive, distressing and controlling (Brophy et al. 2019; Lawn et al. 2015, 2016; Light et al. 2014). Thus, CTOs may conflict with and limit the achievement of key recovery principles, such as ‘assuming control’, ‘managing symptoms’ and ‘becoming empowered and exercising citizenship’. In two qualitative studies conducted in South Australia led by authors with lived experience, consumers reported the CTO experience impacting upon their engagement in the recovery process, describing themselves as being punished for being bad and being seen as untrustworthy (Lawn et al. 2015, 2016). Participants also described lack of engagement with health professionals, reporting that their concerns were dismissed and not listened to (Lawn et al. 2016). Research in NSW and Victoria found consumers describe feeling distress and a loss of autonomy, particularly regarding compulsion to take medication, lack of choice in medications and limited communication about side‐effects (Brophy et al. 2019; Light et al. 2014). Very few participants described being supported to make decisions about their treatment, only having opportunity to participate in decisions when side‐effects or symptoms were unbearable (Brophy et al. 2019). Several Australian researchers have commented on trust playing an important role in whether consumers find CTOs beneficial and that consumer experiences of CTOs depend critically upon the relationship between what is done and how it is done (Brophy et al. 2019; Dawson et al. 2021a; Lawn et al. 2015; McMillan et al. 2019).

McMillan et al. (2019) conducted qualitative research involving people currently on a CTO and mental health professionals, examining the role of trust within the CTO experience in Adelaide, Australia. The authors explored how CTOs produce challenges for creating and maintaining trust and that trust played an important role in whether consumers found their CTO beneficial. The authors discussed the tension in trust forming and functioning within a therapeutic relationship where the fundamental component of the CTO implies the consumer is not trustworthy enough to comply with treatment. Some consumers reported believing the health professional was being open and honest with them, leading to them being able to trust the health professional in part. Conversely, some consumers who did not trust the health professional thought they were not being respected and spoken to honestly and that their viewpoint was not considered important. The study illustrated how consumers and health professionals are aligned in wanting recovery to be an endpoint of a CTO. The authors concluded that ‘for consumers who experienced CTOs as positive and recovery‐oriented, trusting and being trusted by the health professional was a key theme’ (McMillan et al. 2019, 9).

Consumers in both Australian (Carney 2012; Carney and Tait 2011) and international (Macgregor et al. 2019) research have raised concerns regarding their experiences of mental health tribunal hearings where legally binding decisions regarding the continuation of CTOs are made. A Scottish study found half of participants felt the tribunal outcome was a ‘foregone conclusion’ and that expressing their views did not correspond to having increased influence over decision‐making, particularly regarding medications (Ridley and Hunter 2013). Additionally, a systematic review in the United Kingdom found ‘the use of jargon, the inability to challenge evidence as it is presented, being the last person to speak during proceedings and being discouraged from speaking altogether reduces autonomy and disrupts the potential of participating in the tribunal’ (Macgregor et al. 2019, e507). A study in NSW, Victoria and ACT demonstrated that although some consumers value the opportunity to have their voices heard in tribunal hearings, there were limits in what they could discuss (Carney and Tait 2011). Thus, Australian mental health tribunals have been criticised for parochialism, placing a narrow focus on fulfilment of legal criteria without considering wider social and family contexts and medication side‐effects (Carney 2012). This has led to calls in Australian research for greater engagement with consumers' treatment experiences which are often overlooked in tribunal hearings (Carney and Tait 2011).

Although there is a large body of international literature exploring consumers' experiences of CTOs, research led by researchers with lived experience of mental illness has been limited (Brosnan 2018). Consumer perspectives are often entirely absent in academic debates about the merits of CTO use, raising questions about how evidence is created and whose knowledge is omitted from consideration (Brosnan 2018). Researchers with lived experience argue that clinical research dominated by RCTs and systematic reviews is uncritically privileged in academia and marginalises the knowledge and evidence produced by consumer perspectives (Beaupert 2018; Rose 2017). With the current movement towards recovery as a shared goal, symptom management and medication adherence are no longer considered the only measures of effective outcomes, and there is increasing recognition of the need for research that focuses on outcomes that are important to consumers (Rose et al. 2011). Brophy et al. (2018) recommend addressing the paucity of consumer involvement in CTO research by focusing on co‐design and co‐production of interdisciplinary research, inclusive of consumers, families and carers.

### 5.2 Family and Carer Experiences

Compared to studies exploring consumers' and health professionals' perspectives, there are few studies internationally, and even fewer in Australia, reporting on family and carers' experiences of CTOs. A systematic review reported on the findings from 12 qualitative studies in Australia, Canada, New Zealand, Norway, the United States and the United Kingdom exploring the views of families of individuals on a CTO (Corring et al. 2018). The authors found overall families support CTOs but are unhappy with aspects of the process, and CTOs increased families' involvement in care. Families reported benefits of CTOs including systems‐level factors (improved supports from services, health professionals sharing the burden of care with families) (Stensrud, Hoyer, et al. 2015), relational factors (better structure and predictability in the family, reduced risks to caregiver) (Canvin et al. 2014) and clinical improvements (longer periods of wellness, reduced readmissions, improved medication adherence, hope for recovery) (Canvin et al. 2014). Disadvantages included a heavy emphasis on medication as treatment, lack of rigorous follow‐up and overly complex legislative processes (Stensrud, Hoyer, et al. 2015). The international research is similar to findings in two Australian studies conducted in NSW (Light et al. 2014) and Victoria (Vine and Komiti 2015), which found families generally supported CTOs (Vine and Komiti 2015), and CTOs were viewed as a mechanism to secure access to services (Light et al. 2014). In addition, Vine and Komiti (2015) highlighted the apparent high risk of relapse and hospital readmission when the CTO concluded, with the impact and responsibility of this resting with the family.

The experiences of families and carers in mental health tribunal decision‐making and care planning is mixed. Carers hold a variety of roles based on familial and kinship relationships in addition to ‘expert carer’. Carers report experiencing conflict within these differing roles, for example, balancing policing medication adherence with advocating for their loved one who may disagree with treatment (Rugkåsa and Canvin 2017; Stensrud, Hoyer, et al. 2015). Australian studies indicate that involvement in tribunal hearings can lead to disruptions in family dynamics. Families are often asked to provide evidence in tribunal hearings or are involved in instigating involuntary treatment, and thus tensions arise where consumers and families have conflicting views (Carney 2012). In Beaupert and Vernon's (2011) study of carer involvement in care planning and tribunal hearings in NSW, Victoria and ACT, carers reported when they participated, they were listened to, respected and provided opportunity to share their views. However, many carers did not participate, largely because they were not informed about tribunal hearings and because of a lack of transparency regarding their purpose and scope. Rugkåsa and Canvin (2017) highlighted the need for clarifying carer roles and expectations, providing information to support carer involvement, and ensuring continuous, open communication with carers. In summary, families provide most of the caregiving for consumers, and they report that while CTOs reduce their burden of care, there are difficulties with how their voices are included and how CTOs are implemented.

### 5.3 Health Professional Experiences and Approaches to Decision‐Making

Deciding to use involuntary treatment for people living in the community (who do not require hospitalisation) is complex, involving clinical, social, institutional, policy, legal, philosophical and ethical factors (Brophy et al. 2021). While some attempts have been made in Europe to develop decision‐making standards for involuntary hospitalisations (Bartlett 2011; Brissos et al. 2017), far fewer attempts have been made to develop decision‐making standards for initiating and terminating CTOs. The last published attempt to create clinical guidelines for CTOs was in the United States in 1990 (Geller 1990); however, these were based only on the author's clinical experience. In the absence of recent and high‐quality clinical and procedural standards in this area, health professionals are guided by their clinical judgements; however, opinions regarding CTOs are varied and clinical practice reflects this. There is therefore considerable variability in how laws are interpreted and operationalised across jurisdictions, and concerns arise regarding the convenience and availability of legislative rather than evidence‐based approaches, such as assertive community treatment (Kisely and Campbell 2007) and one‐to‐one peer support work (White et al. 2020).

#### 5.3.1 Clinical Factors

Clinical factors associated with CTO use may include an individual's risk of harm to self or others, comorbid substance use and lack of insight leading to treatment nonadherence (Brophy et al. 2019). A recent systematic review across Australia and New Zealand found CTO use was associated with being male, single, unemployed and criminal history (Kisely et al. 2021). In one study of health professional attitudes towards CTOs in Taiwan, the United Kingdom, and New Zealand, participants responded that the usefulness of CTOs was ‘ensuring patients a greater period of stability, ensuring compliance with medication for an extended period and signalling to patients that they have a major mental illness in need of active management’ (Hsieh et al. 2017, 619). In this same study, health professionals reported risk management as the most important factor when deciding to use and terminate CTOs, placing risk management above core clinical considerations, such as ensuring contact with services, rapid relapse management or medication adherence. This finding is echoed in recent Australian research which reported that current practices regarding CTO decision‐making are often guided by service culture and health professionals' attitudes towards coercion and risk management (Dawson et al. 2021b; Light et al. 2017). Few studies include additional criteria of the consumer being likely to deteriorate and experience adverse outcomes because of treatment nonadherence (O'Reilly et al. 2012). In one Norwegian study, a particular dilemma was the difficulty in justifying ongoing coercion if health professionals considered treatment had only partial effect (Stensrud et al. 2016). This has resulted in calls among some academics and health professionals to consider whether the current evidence has enough gravity to support the clinical utility of CTOs (Newton‐Howes and Ryan 2017).

#### 5.3.2 Local Treatment Culture and Staff Attitudes

Australian and international research indicates local treatment culture and staff attitudes are significant determinants in CTO use (Dawson et al. 2021b; Hsieh et al. 2017). Some health professionals, particularly junior psychiatry trainees, report experiencing pressure from supervisors to make coercive decisions they may not agree with or understand (Stensrud et al. 2016). Additionally, a systematic review found that health professionals who encourage risk‐taking or support a consumer's wish to cease medication report fear of blame if negative outcomes result from their decision, leading to the assumption of risk‐averse approaches and restrictive practices (Ahmed et al. 2021). Furthermore, examples of health professionals using reports with out‐of‐date, obsolete, or decontextualised information to demonstrate the risk of violence have been reported in a systematic review (Macgregor et al. 2019).

Health professionals report the level of coercion, concern for human rights and administrative burden are major discouraging factors in using CTOs (Hsieh et al. 2017). In addition, concern for the impact on therapeutic relationships that may drive people away from healthcare services, and the potential shift away from CTOs as a liberty‐enhancing instrument towards their use as a controlling treatment regime, have also been reported by health professionals (Ahmed et al. 2021; Rugkåsa and Burns 2017). Conversely, other studies have shown health professionals relate CTOs to their responsibility to safeguard consumers' long‐term mental health (Stensrud et al. 2016), and it has been suggested that some health professionals mould mental health legislation to fit their own moral beliefs (Foley and Ryan 2020). In a Norwegian qualitative study of staff experiences with CTOs, health professionals were found to justify coercion due to lack of insight, without considering whether consumers could participate in decisions. The authors concluded the participants ‘had a narrow understanding of insight, emphasising the patient's compliance with what clinicians believed was the right approach, more than an assessment of the patient's understanding of his/her own situation’ (Stensrud et al. 2016, 752). These findings are echoed in a recent narrative review which argued that the availability of CTOs as an ‘easy’ alternative to genuine therapeutic engagement with consumers has led to a deskilling in mental health services, which is compounded when CTOs are used to bridge gaps in services, forcing consumers into an overwhelmed system that cannot provide quality services (Maylea et al. 2025).

#### 5.3.3 Systemic Factors and Service System Failures

Research in Australia and New Zealand indicates deficiencies within mental health systems are significant determinants in CTO decision‐making (Brophy et al. 2021; Newton‐Howes and Ryan 2017). Brophy et al. (2021) suggests the possibility that CTOs are used to ensure access to mental health services that may be otherwise unavailable to consumers with severe mental illness. In Stensrud et al.'s (2016) study in Norway, several health professionals reported consumers' finances were taken into consideration when deciding to use a CTO, as consumers received free medications under the CTO and health professionals questioned whether consumers would prioritise medications if they had to pay. It is also suggested that some benefit of CTOs is connected to an operational signal to community mental health services that the consumer requires priority access to healthcare (Newton‐Howes and Ryan 2017). As such, this could encourage consumers and carers to agree with a CTO to access better service provision.

Several mental health acts in Australian jurisdictions make explicit acknowledgement that consumers should not be secluded, restrained, or given medication ‘for reasons of mere administrative or staff convenience’ (*Mental Health Act 2013* (Tas), s. 56). or ‘as an alternative to adequate staffing, equipment or…as a punishment or for the convenience of others, or as a substitute for adequate surveillance, health care professionals, resources or facilities to provide safe care’ (Canberra Health Services 2022, 4). While it is positive that Australian legislation clearly states that some forms of coercive and restrictive practices should not be driven by considerations of convenience, there is a notable absence of extending these provisions towards community‐based coercive interventions such as CTOs. Moreover, the misuse of CTOs to secure better service provision raises other human rights concerns regarding equitable access to healthcare, threatening Article 25 of the UNCPRD ‘the same range, quality and standard of free or affordable health care and programmes as provided to other persons’.

## 6 Relevance for Clinical Practice

It is increasingly recognised in the current landscape that symptom reduction, prevention of hospitalisation and medication adherence are not the only outcome measures of an effective mental health service. With moves towards prioritising recovery principles in mental healthcare, consumer and family perceptions of outcomes and experiences of CTO processes are increasingly gaining recognition in the discourse surrounding CTOs. While significant evidence demonstrates a lack of clinical effectiveness for CTOs, concerns for risk management and gaps in mental health systems contribute to their ongoing use. Mental health professionals experience challenges balancing therapeutic care and concern for human rights with their role enforcing CTOs, partly attributed to a lack of clear guidelines and frameworks for their implementation. This review of the literature suggests the process by which CTOs are implemented has substantial influence on the CTO experience for consumers, families and health professionals. Given the apparent rise in CTO use internationally, future research examining the decision‐making approaches and experiences towards using CTOs and the development of comprehensive guidelines for mental health professionals in their use is needed.

## Conflicts of Interest

The authors declare no conflicts of interest.

## Data Availability

Data sharing is not applicable to this article as no new data were created or analyzed in this study.
